# Collaboration between primitive cell membranes and soluble catalysts

**DOI:** 10.1038/ncomms11041

**Published:** 2016-03-21

**Authors:** Katarzyna P. Adamala, Aaron E. Engelhart, Jack W. Szostak

**Affiliations:** 1 Howard Hughes Medical Institute and Department of Molecular Biology and Center for Computational and Integrative Biology, Massachusetts General Hospital, 185 Cambridge Street, Boston, Massachusetts 02114, USA

## Abstract

One widely held model of early life suggests primitive cells consisted of simple RNA-based catalysts within lipid compartments. One possible selective advantage conferred by an encapsulated catalyst is stabilization of the compartment, resulting from catalyst-promoted synthesis of key membrane components. Here we show model protocell vesicles containing an encapsulated enzyme that promotes the synthesis of simple fatty acid derivatives become stabilized to Mg^2+^, which is required for ribozyme activity and RNA synthesis. Thus, protocells capable of such catalytic transformations would have enjoyed a selective advantage over other protocells in high Mg^2+^ environments. The synthetic transformation requires both the catalyst and vesicles that solubilize the water-insoluble precursor lipid. We suggest that similar modified lipids could have played a key role in early life, and that primitive lipid membranes and encapsulated catalysts, such as ribozymes, may have acted in conjunction with each other, enabling otherwise-impossible chemical transformations within primordial cells.

## Abstract (web summary)

Early cells likely consisted of fatty acid vesicles enclosing magnesium-dependent ribozymes. Here, the authors show that fatty acid derivatives can form vesicles that, unlike those formed from only unmodified fatty acids, are stable in the presence of magnesium and could support ribozyme catalysis.

## Graphical abstract

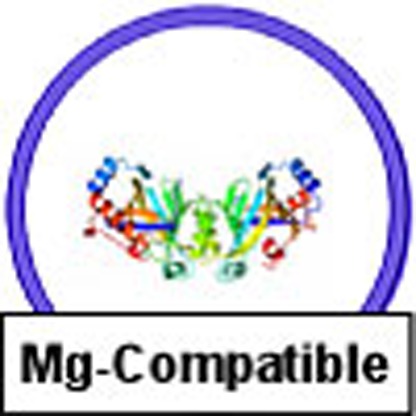

A widely believed model of early life contends that early cellular life was comprised of catalysts and nutrients enclosed within membrane vesicles, much like contemporary cells. The membranes and catalysts found in such a scenario, however, would likely have differed from those observed in extant life, due to the absence of sophisticated protein enzyme catalysts. As a result, simpler alternatives to contemporary biomolecules may have been common in early life. In particular, due to the relative ease of synthesis of single-chain amphiphiles, fatty acid-based membranes, in lieu of the diacylphospholipid membranes largely employed by life today, represent a plausible alternative^1^,^2^,^3^,^4^. Similarly, due to the self-templating capability of nucleic acids, RNA (or a closely related molecule) represents a highly attractive potential polymer for primitive catalysts^5^,^6^,^7^.

Thus, the first protocells could have been comprised of fatty acid membranes and ribozyme catalysts, in contrast to the diacylphospholipid membranes and protein catalysts observed in most contemporary life^5^,^8^. Unfortunately, these substitutions result in another, seemingly paradoxical, problem. Known ribozymes require moderate to high concentrations of divalent cations (typically on the order of 10^−3^ to 10^−2^ M)^9^,^10^. Vesicles composed only of fatty acids, however, are disrupted in the presence of such concentrations of divalent cations due to fatty acid precipitation^11^,^12^. Previously, we demonstrated a potential solution to this problem by employing mixed vesicles containing both a fatty acid and its glycerol ester, which are robust to low millimolar concentrations of Mg^2+^, thus enabling functional encapsulated ribozymes^11^.

While the glycerol ester of a fatty acid represents a partial structural analogue to contemporary diacylphospholipids, esters of carboxylic acids are hydrolytically unstable, and they are subject to ester-amide exchange with free amines. Indeed, ribozymes and simple dipeptide catalysts exist that are capable of promoting the synthesis of amide linkages (including naturally found ones, such as the peptidyl transferase center of the ribosome)^13^,^14^,^15^,^16^. Similarly, the nonenzymatic synthesis of amide linkages by ester-amide exchange with a starting polyester was recently demonstrated^17^. Taken together, these results led us to consider whether an amide analogue of a monoacylglycerol, synthesized by ester-amide exchange, could serve as a membrane component that would confer greater robustness to Mg^2+^ than pure fatty acid vesicles.

Here we show that (1) such a lipid can serve as a membrane component in vesicles, rendering these vesicles robust to millimolar concentrations of magnesium. Thus, (2) these vesicles can support catalysis by an encapsulated ribozyme. Finally, (3) lipid vesicles solubilize a precursor to this lipid amide, which enables an encapsulated catalyst (the protein enzyme α-chymotrypsin, used here as a model substitution for an as-yet undescribed primitive acyltransferase ribozyme) to produce this lipid amide within vesicles. Vesicles containing the catalyst (an enzyme in these experiments) are thereby rendered more robust to Mg^2+^ than those of the same composition lacking the encapsulated catalyst. Furthermore, (4) this conversion is not possible in the absence of vesicles, due to the low solubility of the precursor lipid. In light of these results, we suggest that amide analogues of glycerol esters could have played a role in stabilizing the membranes of early protocells. We also speculate that primitive catalysts and membranes could have worked in concert to enable a wider range of chemical transformations than would otherwise have been possible.

## Results

### Fatty acid derivatives stabilize oleic acid vesicles to Mg^2+^

**Figure 1:**
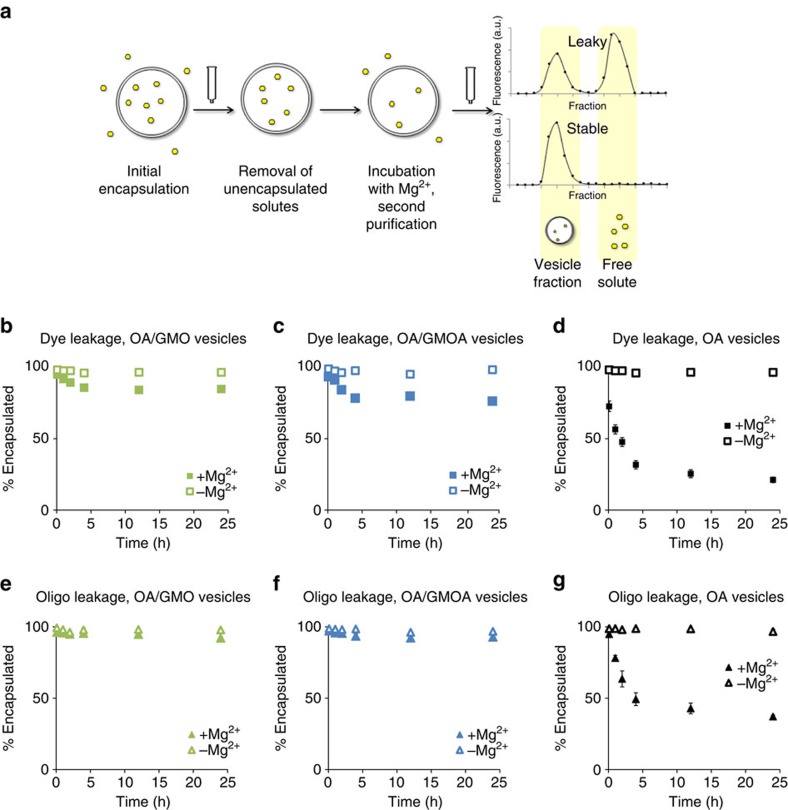
Vesicle leakage measurements. Magnesium-induced leakage of small molecules and oligonucleotides from vesicles of different membrane composition was assayed by encapsulation of the analyte of interest, removal of unencapsulated solutes, addition of magnesium (if applicable), followed by a second purification to quantitate leakage. General scheme of the vesicle stability experiment. (**a**) Columns between panels represent a size-exclusion chromatography step, in which vesicles are separated from unencapsulated solutes. Size-exclusion chromatograms shown here are intended to be schematic in nature only. Leakage of the encapsulated small molecule (calcein (**b**–**d**)) and oligonucleotide (**e**–**g**). Squares: small molecule leakage; triangles: oligonucleotide leakage. Green markers: OA/GMO vesicles; blue markers: OA/GMOA vesicles; black markers: OA vesicles. Error bars are s.e.m. (*n*=4); error bars that are not visible are sufficiently small they are obscured by markers. All reactions contained vesicles comprised of 50 mM total lipid (50 mM OA, or 42.5 mM OA, 7.5 mM (15 mol%) GMO or GMOA), and 0.2 M Na-glycinamide, pH 8.5 as buffer. Mg^2+^-containing samples were 15 mM in Mg^2+^. Calcein, when used, was present at 2.5 mM. Fluorescein-labelled oligonucleotide, when used, was present at 2 μM. Reactions were performed at uncontrolled room temperature (20±2 °C).

We first sought to examine whether a fatty acid glycerol ester (glycerol monooleate, GMO) and its amide analogue (*N-*(2,3-dihydroxypropyl)oleamide, GMOA) were similarly effective in stabilizing oleic acid (OA) vesicles (Fig. 1a). As expected, we observed that pure oleic acid (OA) vesicles (50 mM) containing either an encapsulated small molecule dye (5 mM calcein) or an encapsulated oligonucleotide (2 μM fluorescein-labelled DNA 9mer) readily leaked in the presence of 10 mM Mg^2+^, with only 21% retention of dye and 37% retention of oligonucleotide after 24 h (Fig. 1d,g). The presence of 15 mol% GMO or GMOA, in each case, stabilized vesicles substantially to magnesium: OA/GMO vesicles retained 85% of dye and 92% of oligonucleotide after 24 h (Fig. 1b,e), while OA/GMOA vesicles gave comparable performance, retaining 76% of dye and 93% of oligonucleotide after 24 h (Fig. 1c,f). Given that oligonucleotide permeability of fatty acid liposomes is highly length dependent, with longer oligonucleotides being markedly better retained than shorter ones^18^, these vesicles are anticipated to retain longer oligonucleotides (for example, ribozymes, as employed below) as well or better than the short 9 nucleotide oligomers employed in the leakage study described.

### Vesicles containing GMO(A) support ribozyme catalysis

Retention of encapsulated oligonucleotides in the presence of divalent cations is a critical feature of a protocell membrane. This, alone, is not sufficient, however, as most ribozymes require free (that is, unchelated) Mg^2+^. Having verified that GMOA was as effective as GMO in enabling OA vesicles to retain their contents in the presence of Mg^2+^, we next investigated whether OA/GMO and OA/GMOA vesicles are (1) permeable to Mg^2+^ and (2) contain free internal Mg^2+^ when incubated in solutions containing Mg^2+^ ions. To do so, we employed mag-fura-2, a vesicle-impermeable dye which gives a ratiometric fluorescence response to free Mg^2+^ (ref. 11). We monitored the transit of Mg^2+^ across the membrane of OA/GMO and OA/GMOA vesicles containing mag-fura-2, using a calibration curve of mag-fura-2 with free Mg^2+^ (Supplementary Figs 1 and 2). For both vesicle compositions, Mg^2+^ equilibration across the membrane was rapid and complete in ca. 2 min. Consistent with the longer acyl chain length, this equilibration took somewhat longer than the 20 s required for the shorter chain myristoleic acid/glycerol monomyristoleate (MA/GMM) vesicles previously studied^11^.

**Figure 2:**
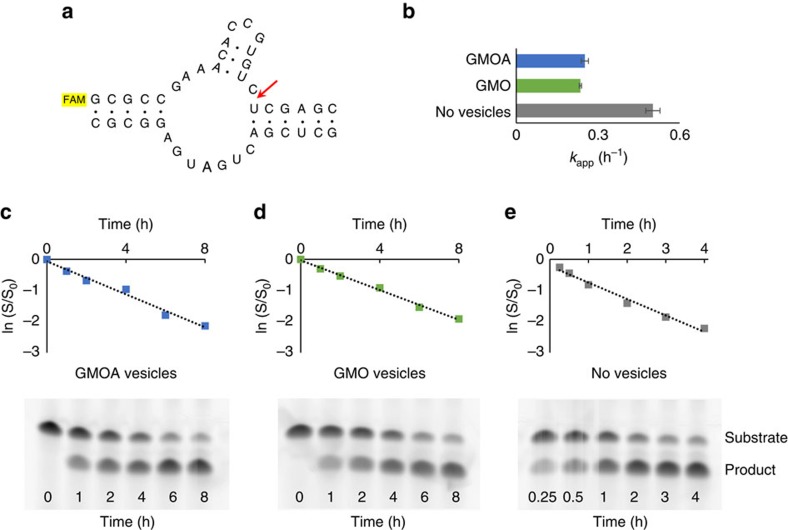
OA/GMOA and OA/GMO vesicles support ribozyme activity. Fluorescein-labelled hammerhead ribozymes (**a**) were encapsulated within OA/GMOA- and OA/GMO-containing vesicles. Cleavage rates for this ribozyme were measured in both types of vesicles as well as in the absence of vesicles. At 15 mM Mg^2+^, OA/GMOA vesicles gave similar ribozyme cleavage rates to OA/GMO-containing vesicles (**b**–**e**) Green markers: OA/GMO vesicles, *k*_app_=0.24 h^−1^ (s.e.m.=0.004); blue markers: OA/GMOA vesicles, *k*_app_=0.25 h^−1^ (s.e.m.=0.013); grey markers: no vesicles, *k*_app_=0.50 h^−1^ (s.e.m.=0.026). Representative polyacrylamide gel electrophoresis (PAGE) analyses of hammerhead ribozyme reactions for each set of vesicle conditions are shown below each graph. Error bars on **b** are s.e.m. (*n*=3). Solid lines on **c**,**d** and **e** are linear fits; for all, *R*^2^>0.98. All reactions contained vesicles comprised of 50 mM total lipid (50 mM OA, or 42.5 mM OA, 7.5 mM (15 mol%) GMO or GMOA), and 0.2 M Na-glycinamide, pH 8.5 as buffer, RNA concentration was 1 μM of each hammerhead strand. Mg^2+^ was present at 15 mM. Reactions were performed at uncontrolled room temperature (20±2 °C).

Having ascertained that Mg^2+^ transit across OA/GMOA membranes was rapid and free Mg^2+^ was present within these vesicles, we examined the functionality of an encapsulated hammerhead ribozyme^19^,^20^. This ribozyme exhibits Mg^2+^-promoted catalytic function. Consistent with the presence of free Mg^2+^ within these vesicles, a reaction containing 1 μM of each ribozyme component and 15 mM MgCl_2_ exhibited a rate constant of 0.24 h^−1^ in OA/GMO vesicles and 0.25 h^−1^ in OA/GMOA vesicles—comparable between lipid systems and somewhat lower than the vesicle-free rate constant of 0.50 h^−1^ (Fig. 2).

### Vesicles with a GMOA synthesis catalyst are Mg^2+^ stabilized

**Figure 3:**
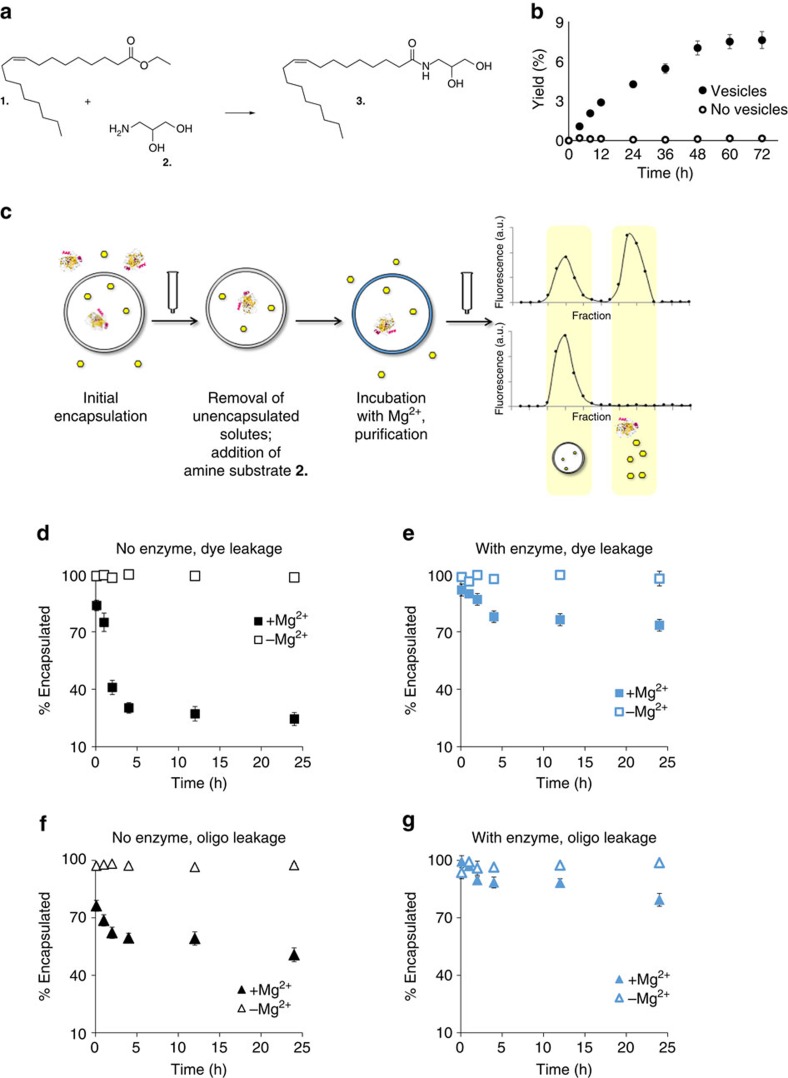
Encapsulated α-chymotrypsin generates GMOA *in situ* and renders vesicles robust to Mg^2+^. General scheme of the experiment (**a**). Vesicles containing ethyl oleate within the membrane and α-chymotrypsin within their lumen, in the presence of (±)-3-amino-1,2-propanediol, convert these substrates to GMOA. α-chymotrypsin-catalyzed conversion of ethyl oleate to GMOA requires the presence of vesicles, which solubilize the precursor lipid. (**b**) OA/GMOA vesicles, derived from α-chymotrypsin-catalyzed conversion of ethyl oleate after 72 h incubation, are stabilized to Mg^2+^-induced leakage. (**d**–**g**) Analysis of vesicles by repurification following Mg^2+^ exposure (general scheme of the assay on **c**; size-exclusion chromatograms shown here are intended to be schematic in nature only) revealed that *in situ* generated GMOA dramatically reduced Mg^2+^-induced leakage of small molecule dye and oligonucleotides from α-chymotrypsin-containing vesicles. (**d**–**g**) Leakage of the encapsulated small molecule (calcein; **d**,**e**) and oligonucleotide (**f**,**g**). Squares: small molecule leakage; triangles: oligonucleotide leakage. Blue markers: vesicles with the enzyme, black markers: vesicles without the enzyme. Error bars are s.e.m. (*n*=4); error bars that are not visible are sufficiently small they are obscured by markers. All reactions contained vesicles comprised of 50 mM total lipid (42.5 mM OA, 7.5 mM (15 mol%) EtOA), 25 mM (±)-3-amino-1,2-propandiol, and 0.2 M Na-glycinamide buffer, pH 8.5 as buffer. α-chymotrypsin, when present, was 1 mg ml^−1^ (within vesicles). Reactions were performed at uncontrolled room temperature (20±2 °C).

As a model of a prebiotic liposomal system, we encapsulated α-chymotrypsin within vesicles comprised of oleic acid and ethyl oleate, in the presence of (±)-3-amino-1,2-propanediol. As this enzyme is known to convert hydrophobic ethyl esters to amides^21^, we reasoned that the α-chymotrypsin-catalyzed formation of GMOA from the ethyl oleate precursor would result in vesicles that were more robust to Mg^2+^. The conversion of ethyl oleate to GMOA in this system was efficient, with ca. 70% yield over 72 h (Fig. 3), corresponding to ca. 10 mol% GMOA. We tested these post-incubation vesicles for robustness to Mg^2+^, and observed that they were significantly more effective at retaining their contents, with 68% retention of calcein and 87% retention of oligonucleotide over 24 h of incubation with Mg^2+^ (cf. 19% dye retention and 52% oligonucleotide retention for vesicles of the same composition without encapsulated α-chymotrypsin). These results were similar to those observed in vesicles formed with nonenzymatically synthesized GMOA (Fig. 1, example purification traces are shown in Supplementary Figs 3 and 4).

### Vesicles are required for enzymatic GMOA synthesis

In addition to the catalytic impact of α-chymotrypsin, we observed that the vesicles, themselves, are promoters of the transformation of ethyl oleate to GMOA. When we attempted to perform the same enzymatic reaction in aqueous solution lacking liposomes, essentially no conversion occurred, at least in part due to the negligible solubility of ethyl oleate (Fig. 3b). Thus, we suggest that the vesicle membrane itself is a phase-transfer catalyst of this transformation, due to its ability to solubilize ethyl oleate, as well as potential favourable interactions between cationic (±)-3-amino-1,2-propanediol and the negatively charged fatty acid bilayer.

## Discussion

We have shown that α-chymotrypsin can act as a catalyst within model protocell vesicles comprised of OA and ethyl oleate, enabling the *in situ* generation of the lipid GMOA. In turn, GMOA is incorporated into the vesicle membranes, thus rendering these vesicles robust to the presence of divalent cations—an absolute requirement for an RNA-based form of primitive metabolism^9^,^22^,^23^. The high level of retention of oligonucleotides enabled by this system, while simultaneously allowing for some small molecule permeability, would have been a highly adaptive trait for a primitive cell, enabling greater retention of catalysts (such as ribozymes) than otherwise possible, while allowing for permeation of nutrients (such as nucleotide monomers)^24^,^25^.

These results amount to a primitive adaptive behaviour for a compartment, driven by an encapsulated catalyst. While α-chymotrypsin, the catalyst employed in this work, is not a prebiotic enzyme, we note that several ribozymes exist that catalyse the formation of amide linkages, suggesting that the transformations performed in this work are within the catalytic repertoire of RNA^26^,^27^,^28^. Furthermore, given the enhanced hydrolytic stability of amides relative to esters, compounds such as GMOA would have been more stable in the presence of divalent metal ions and more robust to heating and wet/dry cycles on the early earth, as was recently observed in the conversion of esters to depsipeptides^17^.

Our observation that the presence of vesicles is essential for the conversion of ethyl oleate to GMOA is fully consistent with previous suggestions that fatty acid membranes themselves could have functioned as reaction promoters in early life^29^. Indeed, before the advent of coded protein synthesis, it is likely that such interactions were crucial. Contemporary life employs proteins that fold with exquisite specificity to form hydrophobic cores capable of binding water-insoluble substrates. It would be more difficult for the highly charged RNA polymer to have played such a role. Fatty acid membranes could have played a key role in solubilizing such molecules and enabling them to undergo chemical transformations that were otherwise inaccessible in aqueous solution. We speculate that scenarios in early life in which membranes and soluble catalysts collaborated could have been critical to early life. Ribozyme catalysts could have enabled reactions of higher specificity than a simple self-assembled membrane bilayer, and the lipid bilayer could have solubilized components that otherwise would not be available for reaction in bulk aqueous solution.

Here, we have shown that GMOA, the amide analogue of GMO, is as effective as GMO in stabilizing lipid vesicles against divalent ion-mediated lysis and precipitation, and vesicles containing either lipid can support ribozyme catalysis. The comparative hydrolytic stability of GMOA relative to GMO leads us to speculate that GMOA (and related amino-sugar-based fatty acid amides) could have served as membrane-stabilizing lipids before the emergence of coded protein catalysts.

Recently, several other groups have observed that lipid assemblies can bind biopolymer building blocks^30^ and modulate DNA aptamer specificity^31^. Our results here demonstrate that the functional repertoire of lipids extends yet further, into the realm of enabling soluble catalysts to promote reactions that are otherwise disfavoured or impossible in aqueous solution. Our observation that both a catalyst and a fatty acid-based membrane acting in concert were required for the aqueous synthesis of GMOA suggests that lipids and catalysts may have coevolved in early life, with soluble fatty acids serving to bring hydrophobic reactants into solution in primitive encapsulated catalyst systems. As more sophisticated proteins with hydrophobic cores evolved, this crude solubilization role would have been supplanted by the catalysts themselves. In turn, less soluble lipids capable of forming more stable membranes could have supplanted more soluble fatty acids. Early forms of life comprised principally of ribozyme/fatty acid-based systems could, thus, have evolved in concert into the coded protein catalyst/diacylphospholipid system that predominates in life today.

## Methods

### Suppliers

Unless otherwise stated, all chemicals and enzymes were purchased from Sigma-Aldrich Corp. (St Louis, MO, USA) and used without further purification.

### Preparation of vesicles

Oleic acid/oleate (OA) vesicles were prepared by resuspending oleic acid in buffer (0.2 M Na-glycinamide pH 8.5), and then sonicating for about 1 min, followed by five freeze-thaw cycles. After that, samples were typically left tumbling for 24 h and then extruded through a 100 nm filter using a Mini-Extruder system (Avanti Polar Lipids, Inc., Alabaster, AL, USA), as previously described^11^. Glycinamide buffer was used in place of the more common bicine buffer because it is more prebiotically plausible while simultaneously exhibiting a similar p*K*_a_ and membrane permeability to bicine. *N*-oleoylglycinamide (NOG), the hypothetical amide condensation product of ethyl oleate and glycinamide, was not observed in reactions (Supplementary Fig. 5). NOG prepared by chemical synthesis could form vesicles in combination with oleic acid, but these vesicles leaked in the presence of magnesium (Supplementary Fig. 6). For vesicles prepared with encapsulated enzyme and dye (or dye-labelled oligonucleotide), the enzyme and dye-containing component were dissolved in buffer prior to addition of lipid, and vesicles were then treated as described above. After extrusion, vesicles were left tumbling to equilibrate for at least 2 h. Vesicles were purified from unencapsulated solutes on a gravity-flow size-exclusion column (Sepharose 4B, wet bead diameter 45–165 μm) with glycinamide buffer as the mobile phase. The vesicles, with an average diameter of 100 nm after extrusion, elute with the void volume of the column, followed by unencapsulated enzyme (30–60 kDa) and calcein dye (<1 kDa) or oligonucleotide (ca. 3 kDa).

Mixed composition vesicles, made of oleic acid and ethyl oleate (EtOA) or GMO or GMOA or NOG, were prepared by mixing oleic acid with a chloroform or dichloromethane solution of EtOA or GMO or GMOA or NOG, removing the solvent on a rotary evaporator, and then resuspending the mixture in buffer with or without solutes, after which the vesicles were prepared as described above. In each case, the coelution of encapsulated dye or dye-labelled oligonucleotide with the vesicle fraction during gel purification confirmed encapsulation and the presence of vesicles (versus oil droplets, which would not encapsulate solutes). Vesicles were 50 mM total lipid; where a second lipid component was present, it comprised 15 mol% of total lipid (that is, 42.5 mM OA, 7.5 mM EtOA, GMO, GMOA or NOG).

### α-Chymotrypsin activity in vesicles

For α-chymotrypsin reactions, vesicles comprised of OA and EtOA with encapsulated α-chymotrypsin and calcein were prepared as described above and incubated in the presence of 25 mM glycerol amine analogue (±)-3-amino-1,2-propanediol. The pH of mixture of calcein and (±)-3-amino-1,2-propanediol in the glycinamide buffer was adjusted to 8.5 before the addition of the enzyme and lipids. The initial concentration of α-chymotrypsin was 1 mg ml^−1^; vesicles were 50 mM total lipid (42.5 mM OA, 7.5 mM (15 mol%) EtOA). The buffer employed was 0.2 M Na-glycinamide buffer, pH 8.5. In a typical experiment, OA/EtOA vesicles were prepared with α-chymotrypsin in the solution, followed by equilibration, extrusion and removal of unencapsulated solutes. (±)-3-amino-1,2-propanediol was then added and samples were left to tumble at 37 °C for 72 h. The p*K*_a_ of the conjugate acid of (±)-3-amino-1,2-propanediol was determined to be 9.18 (Supplementary Fig. 7), indicating that, in bulk solution, 17% of this substance was present as the free base.

The reaction yield was calculated from the ^13^C NMR spectrum (Varian VXR-400). Samples were prepared as follows: at a given time point, a sample was frozen in liquid nitrogen, then lyophilized; the lipid compounds were redissolved in a small volume of CDCl_3_. Redissolution in CDCl_3_ was employed to dissociate vesicles, thus avoiding the extensive line broadening due to the slow tumbling of large vesicles. A representative ^13^C NMR of the carbonyl region of a GMOA/EtOA mixture and the ^13^C NMR spectrum of GMOA are given in Supplementary Fig. 5. The yield was calculated by referencing a ratio of the heights of the 178.8 p.p.m. peak corresponding to the ester carbonyl of EtOA and the 174.5 p.p.m. peak corresponding to the amide carbonyl of GMOA to a calibration curve (Supplementary Fig. 8). As both these resonances correspond to carbonyl carbons, we anticipated that intensity variations due to proton decoupling could be neglected. To validate this, control experiments with a CDCl_3_ solution containing known quantities of EtOA and GMOA were performed; these gave the same ratiometric quantitation results as a CDCl_3_ extract of the lyophilization residue of an aqueous sample of the same composition.

### Synthesis of GMOA

GMOA was synthesized and purified as described previously^32^. Oleic acid (0.159 ml, 0.141 g, 0.5 mmol) was dissolved in 40 ml of dry CH_2_Cl_2_. *N*-(3-dimethylaminopropyl)-*N*′-ethylcarbodiimide hydrochloride in the measure 0.326 g (2 mmol) was added, followed by 0.279 ml (2 mmol) triethylamine and a catalytic amount of DMAP; after 10 min of vigorous stirring, 0.116 ml (1.5 mmol) of (±)-3-amino-1,2-propanediol was added. The reaction was stirred at RT for 5 h. The solvent was removed by rotary evaporation, and the resulting colourless oil was purified on a silica gel flash column with a hexane:AcOEt gradient (2:8 to 7:3). GMOA in the measure 0.146 g (0.41 mmol, 82% yield) was obtained as a colourless oil which solidified on standing. Electrospray ionization-mass spectrometry (ESI-MS): Calculated *m*/*z* for C_21_H_42_NO_3_^+^ [M+H]: 356.3; observed: 356.2. ^1^H NMR (CDCl_3_, 400 MHz): 0.87 (t, 3H), 1.26 (m, 20H), 1.59 (m, 2H), 2.15 (m, 6H), 3.38 (m, 2H), 3.59 (m, 2H), 3.70 (br, 2H), 3.98 (m, 1H), 5.41 (m, 2H), 7.21 (br, 1H). ^13^C NMR (CDCl_3_, 100 MHz): 14.12, 22.71, 25.78, 28.63, 29.05, 29.11, 29.65, 29.69, 29.74, 29.82, 31.94, 34.12, 36.52, 43.90, 65.06, 71.24, 131.56, 131.60 and 174.51.

### Synthesis of NOG

Oleic acid (947 μl, 3 mmol), N, N, N′, N′ - tetramethyl-0-(1H-benzotriazol-1-y1) urcnium hexafluorophosphate (HBTU) (1138, mg, 3 mmol) and diisopropylethylamine (2613 μl, 15 mmol) were stirred in 75 ml dichloromethane for 1 h at room temperature. Glycinamide hydrochloride (365 mg, 3.3 mmol) was then added, and the resulting mixture was stirred overnight at room temperature. The reaction was filtered, and the filtrate was concentrated by rotary evaporation onto 5 g of silica. The reaction was then dry-loaded and purified on a silica gel flash column with a EtOAc:MeOH gradient (1:0 to 9:1). The compound eluted as a single peak comprised of NOG·3.5 N,N-Diisopropylethylamine DIEA) (by ^1^H NMR integration), of which 570 mg (0.72 mmol, 24% yield based on overall molar mass of the mixture) was obtained as a white solid. Recrystallization of 150 mg of this mixture from ca. 30 ml ethyl acetate yielded 33 mg of NOG containing no detectable DIEA (51% recovery). Electrospray ionization-mass spectrometry (ESI-MS): Calculated *m*/*z* for C_20_H_38_N_2_O_2_Na^+^ [M+Na]: 361.5; observed: 361.1. ^1^H NMR (Methanol-d4, 400 MHz): 0.92 (t, 3H), 1.34 (m, 20H), 1.64 (p, 2H), 2.05 (m, 4H), 2.28 (t, 2H), 3.85 (br s, 2H), 5.36 (m, 2H). ^13^C NMR (CDCl_3_, 100 MHz): 14.30, 22.87, 25.76, 27.36, 27.41, 29.31, 29.42, 29.51, 29.71, 29.89, 29.95, 32.09, 36.53, 43.06, 129.90, 130.21, 171.29, 174.07. These spectral data are consistent with those previously reported for this compound^33^.

### Vesicle leakage after magnesium exposure

Vesicle stability was assessed by measuring the leakage of either an encapsulated small molecule dye (calcein) or a dye-labelled oligonucleotide versus time. After vesicle formation, unencapsulated solute (2.5 mM calcein or 2 μM 5′-d(CCA ATG CGC)-3′-fluorescein) was removed by size-exclusion chromatography (Sepharose 4B), with running buffer containing the same concentration and composition of lipids as the vesicle sample.

In a typical experiment, a 3 ml purified vesicle sample was mixed with 0.2 ml of MgCl_2_ solution (240 mM, to give the desired final Mg^2+^ concentration of 15 mM) while stirring rapidly to minimize vesicle disruption by transient exposure of vesicles to high magnesium concentrations. We observed that the rapid mixing of magnesium is critical to vesicle stability. If magnesium is added and vesicles are not immediately vigorously vortexed, permeability experiments produce inconsistent results from sample to sample. After Mg^2+^ addition, the sample was allowed to tumble for the given time and repurified on a size-exclusion column, as described above.

Calcein or oligonucleotide label fluorescence was recorded using a SpectraMax Gemini EM plate reader (Molecular Devices). Vesicle samples were diluted to ca. 1 mM lipid to minimize fluorescence artifacts arising from scattering. The total fluorescence of all vesicle and all free dye/oligonucleotide fractions was calculated; leakage is reported as % leakage=(*F*_dye_ × 100%)/(*F*_ves_+*F*_dye_), where *F*_ves_ is total fluorescence of all vesicle fractions and *F*_dye_ is total fluorescence of all unencapsulated dye/oligonucleotide fractions collected during the size-exclusion column purification. The initial calcein concentration was 2.5 mM; at this concentration, self-quenching is negligible.

Magnesium exposure experiments presented in the main text were performed in four replicates, independently mixing samples with magnesium and purifying on the column. Reported leakage values are the arithmetic average of four experiments. The reported error bars are s.e.m. (*n*=4).

### Mag-fura-2 assay for free Mg^2+^ measurement

The permeability assays were performed as described previously^11^. Briefly, vesicles were prepared as described above, with mag-fura-2 tetrapotassium salt encapsulated inside vesicles at a concentration of 2.5 mM. Free dye was removed by size-exclusion column chromatography. Vesicles were adjusted to ca. 5 mM total lipid, and Mg^2+^ was added as a stock of MgCl_2_ to a final concentration of 2.5 mM.

The concentration of free magnesium inside vesicles was calculated by fitting the measured fluorescence ratio of fluorescence emission measured with (*λ*_em_=500 nm, *λ*_ex_= 340 nm) to that measured with *λ*_ex_=370 nm, *λ*_em_=500 nm to a standard calibration curve of known Mg^2+^ (as MgCl_2_) concentration (Supplementary Fig. 2).

## Additional information

**How to cite this article**: Adamala, K. P. *et al.* Collaboration between primitive cell membranes and soluble catalysts. *Nat. Commun.* 7:11041 doi: 10.1038/ncomms11041 (2016).

## Supplementary Material

Supplementary Information Supplementary Figures 1-8
