# Supplementary material for: Collaboration between primitive cell membranes and soluble catalysts
Source: Nat Commun. 2016 Mar 21;7:11041. doi: 10.1038/ncomms11041 (PMC4802160; doi:10.1038/ncomms11041)
Supplement: Supplementary Information — Supplementary Figures 1-8 [file ncomms11041-s1.pdf]

a

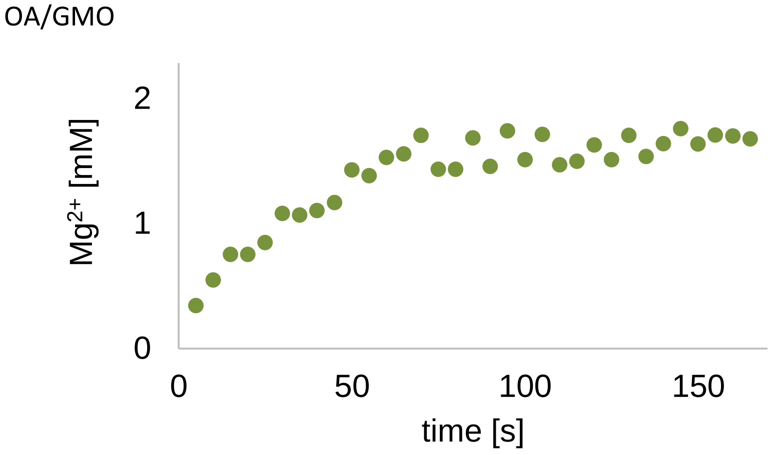

b

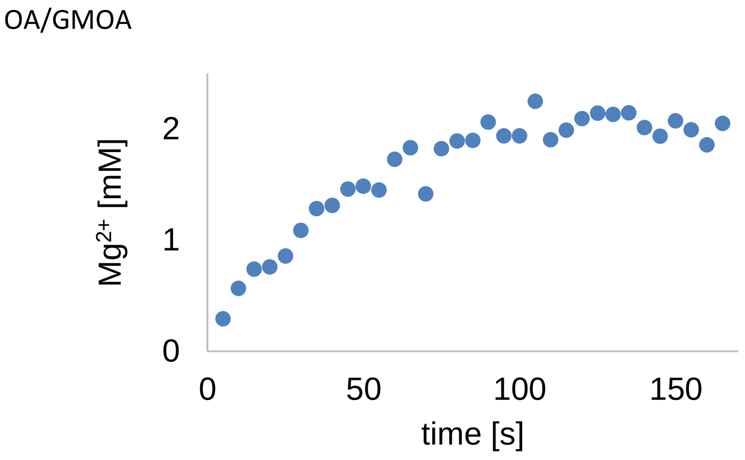

Supplementary Figure 1. Magnesium permeability through OA/GMO and OA/GMOA vesicle membranes. Free Mg^{2+} concentrations were measured using mag-fura-2, using a standard calibration curve (Supplementary Figure 2). Mag-fura-2 was present at a concentration of 2.5 mM.

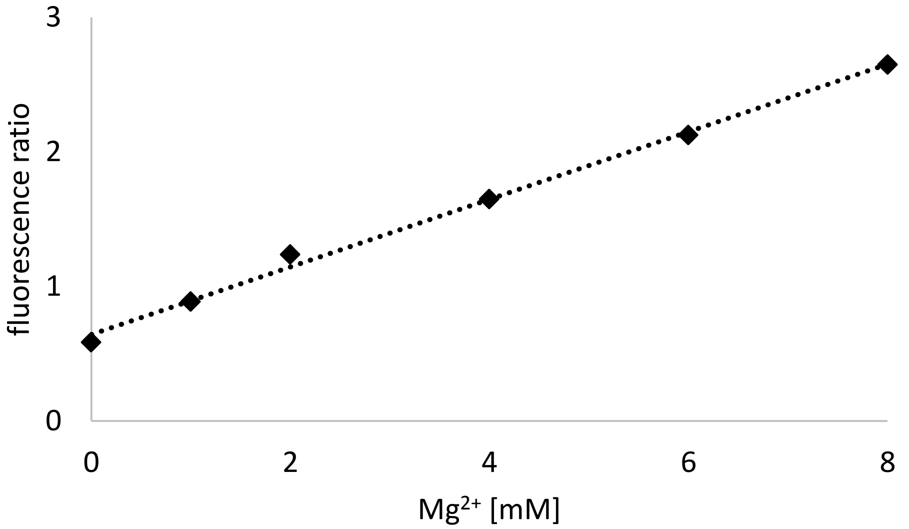

Supplementary Figure 2. Mag-fura-2 fluorescence calibration curve for free Mg^{2+}.

The value reported is the ratio of fluorescence emission measured with (λ_{em}=500 nm, λ_{ex}= 340 nm) to that measured with λ_{ex}=370 nm, λ_{em}=500 nm. Mag-fura-2 was present at a concentration of 2.5 mM Dotted line is linear fit, R^2=0.99.

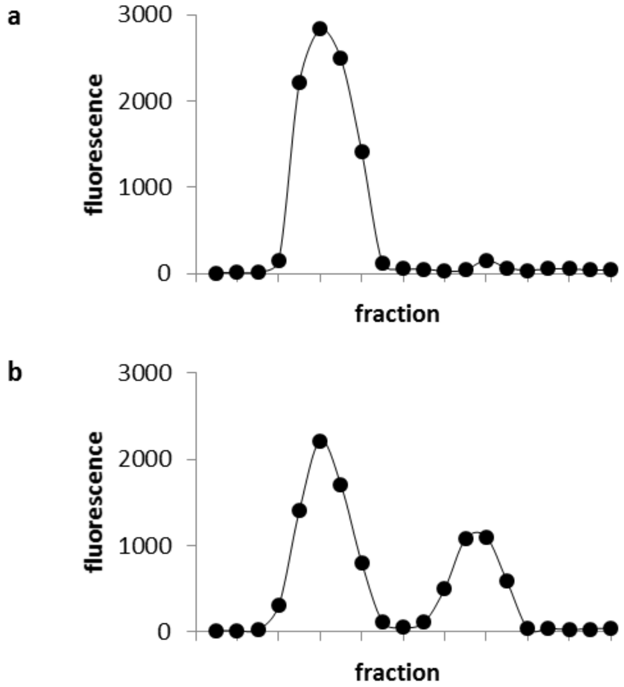

Supplementary Figure 3. Example size exclusion column purification traces for the leakage of a small molecule dye (calcein) encapsulated inside vesicles at a concentration of 2.5 mM.

First peak is vesicles with encapsulated dye, second peak is free dye.

A: OA/GMOA vesicles, 4 h incubation with 10 mM Mg^{2+}.

B: Pure OA vesicles, exposed to 10 mM Mg^{2+} and immediately purified.

Solid lines connecting the points are optical guides, not fits.

The measurements were taken with λ_{ex}=495 nm and λ_{em}= 520 nm.

Chromatography was performed in 1.5x12cm diameter columns, under gravitational flow. Each collected fraction is approximately 0.2-0.25ml (the automated fraction collector used in those experiments counts droplets, not volume).

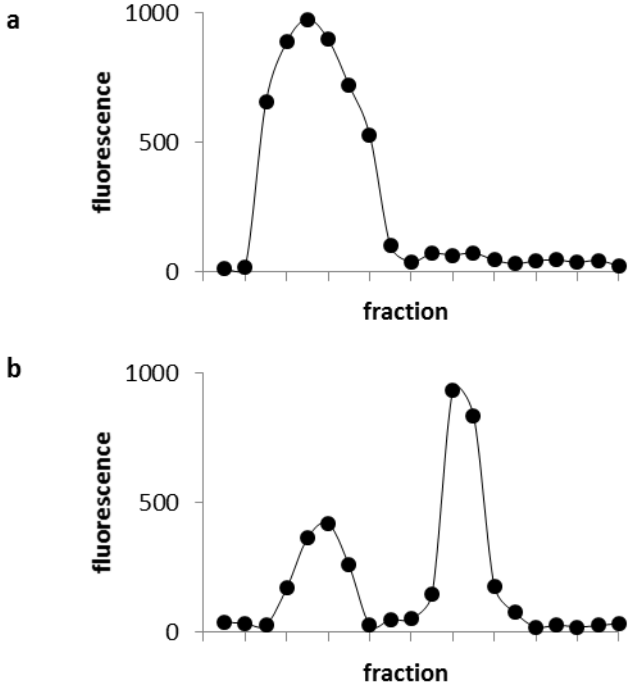

Supplementary Figure 4. Example size exclusion column purification traces for the leakage of an oligonucleotide (5′-d(CCA ATG CGC)-3′-fluorescein), encapsulated inside vesicles at a concentration of 2 µM.

The first peak is vesicles with encapsulated solute, and the second peak is free oligonucleotide.

A: OA/GMO vesicles exposed to 10 mM Mg^{2+} for 24 h.

B: Pure OA vesicles, exposed to 10 mM Mg^{2+} for 12 h.

Solid lines connecting the points are optical guides, not fits.

The measurements were taken with λ_{ex}=495 nm and λ_{em}= 520 nm.

Chromatography was performed in 1.5x12cm diameter columns, under gravitational flow. Each collected fraction is approximately 0.2-0.25ml (the automated fraction collector used in those experiments counts droplets, not volume).

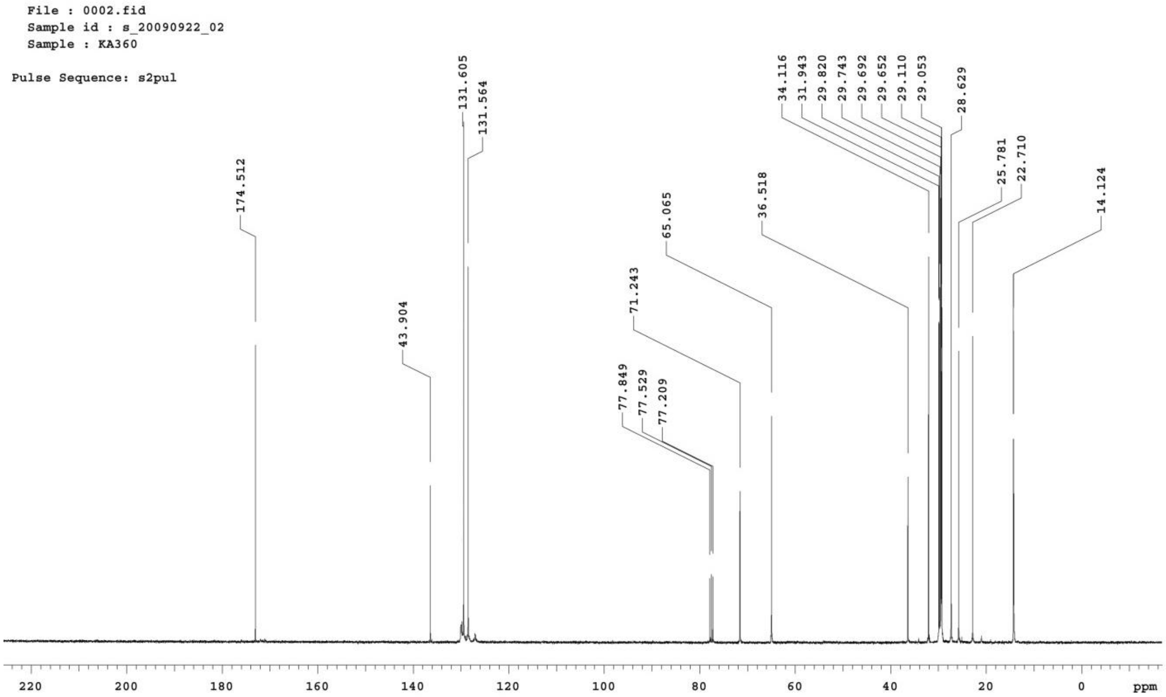
File : 0002.fid
Sample id : s_20090922_02
Sample : KA360
Pulse Sequence: s2pul
174.512
43.904
131.605
131.564
77.849
77.529
77.209
71.243
65.065
36.518
34.116
31.943
29.820
29.743
29.692
29.652
29.110
29.053
28.629
25.781
22.710
14.124
220
200
180
160
140
120
100
80
60
40
20
0
ppm

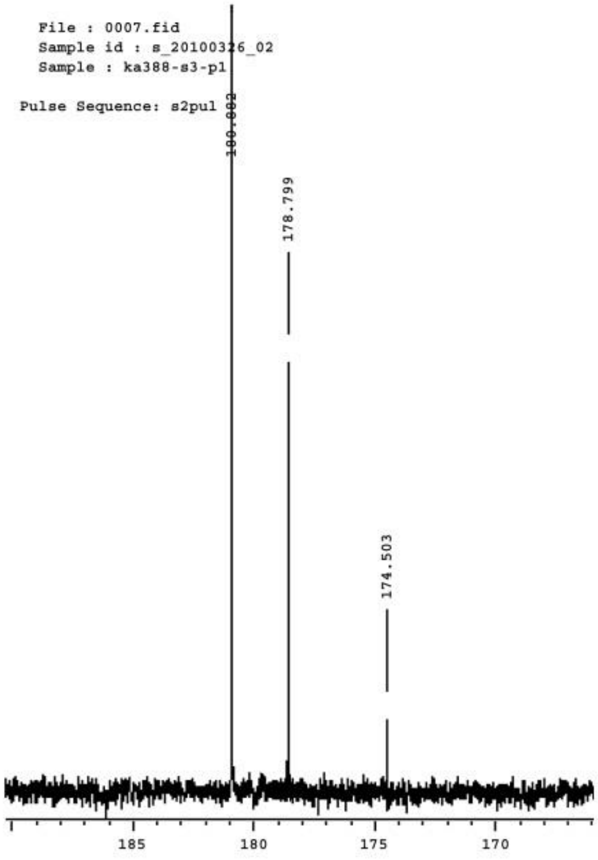
File : 0007.fid
Sample id : s_20100326_02
Sample : ka388-s3-p1
Pulse Sequence: s2pul
178.799
174.503
185
180
175
170

Supplementary Figure 5. ^{13}C NMR spectra of GMOA (top) and lyophilized liposomes containing GMOA-generating enzymatic reaction components (bottom). Carbonyl resonances for GMOA were observed at 174.5 ppm, EtOA at 178.8 ppm, and oleic acid/oleate at 180.8 ppm. Resonances for NOG (174.1 ppm and 171.3 ppm) were not observed. Spectra were obtained in $CDCl_3$.

a

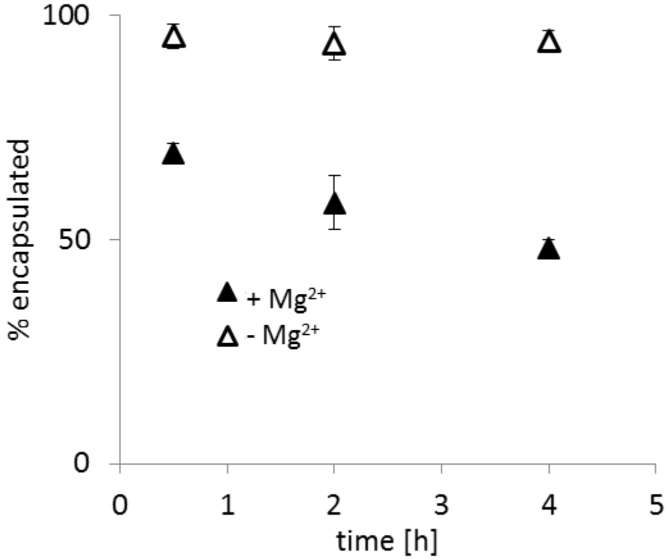

b

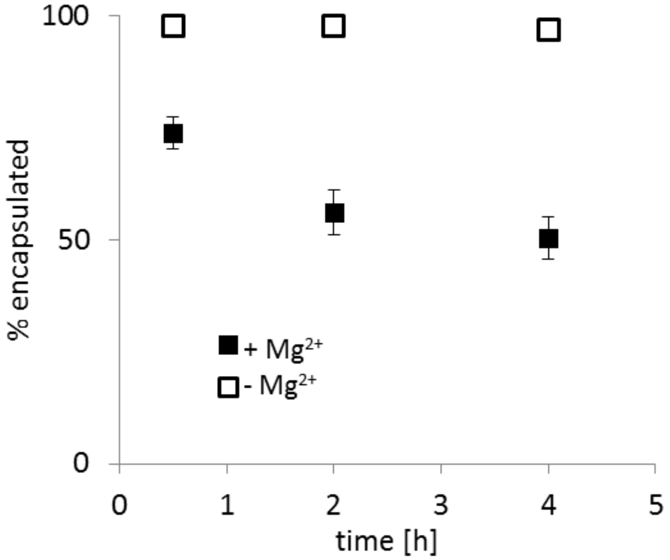

Supplementary Figure 6. Magnesium-induced leakage of OA/NOG vesicles. Vesicles (85 mol% OA, 15 mol% NOG) containing dye (**a,** top) or oligonucleotide (**b**, bottom) were subjected to magnesium leakage assays as described in the Methods section. Vesicles of this lipid composition could retain dye (**a**) or oligonucleotide (**b**) but exhibited rapid leakage of both in the presence of Mg^{2+}. Error bars represent S.E.M., N=3; some error bars are sufficiently small they are obscured by markers.

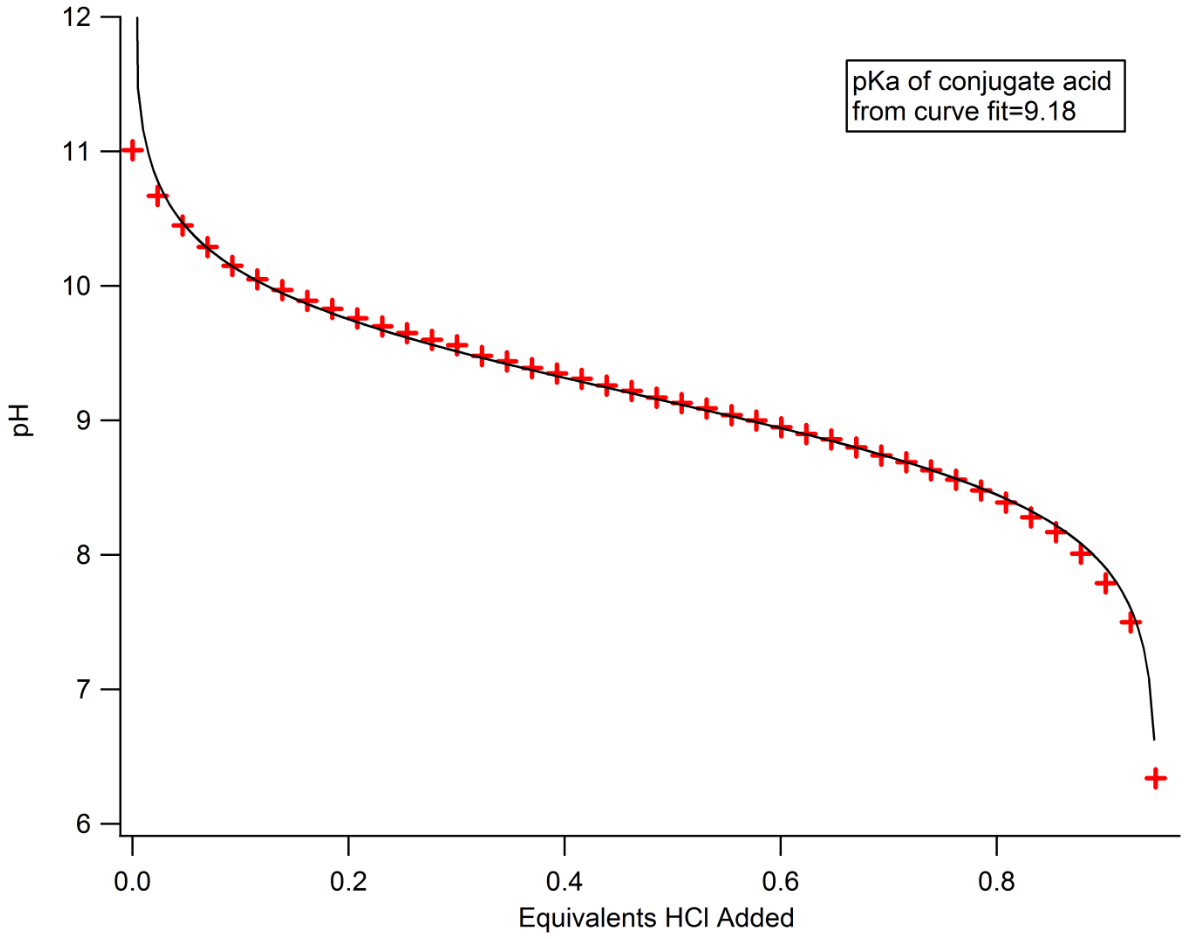

Supplementary Figure 7. pKa determination of conjugate acid of (±)-3-amino-1,2-propanediol. 201 mg (2.21 mmol) (±)-3-amino-propane-1,2-diol in an initial volume of ca. 15 mL water was titrated with 0.5097 N HCl (aq.) in 100 µL portions (red crosses). Fitting the resulting data to the Henderson-Hasselbalch equation (black trace) gave a pKa for the conjugate acid of this compound of 9.18.

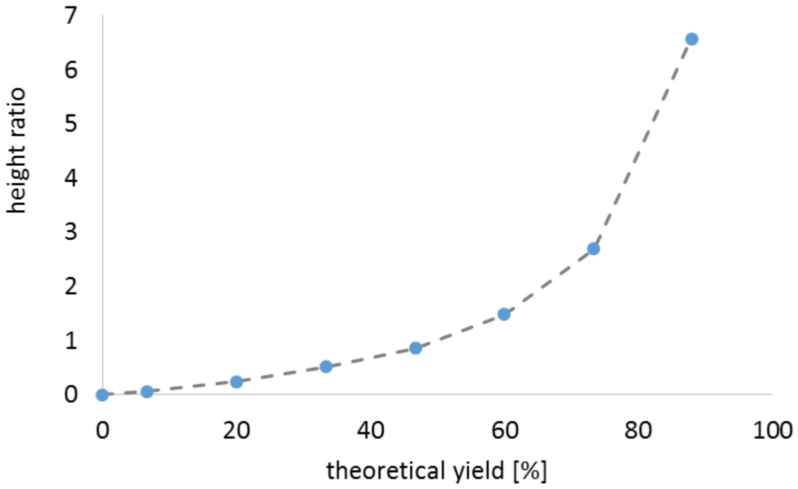

Supplementary Figure 8. ^{13}C NMR yield calibration curve for ethyl oleate and GMOA mixtures. Samples were prepared by mixing pure GMOA and EtOA. Height ratios were obtained by obtaining a ^{13}C NMR spectrum of these mixtures. Height ratio is reported as the ratio of the height of the GMOA amide carbonyl peak (174.5 ppm) to that of the EtOA ester carbonyl peak (178.8 ppm). Theoretical yield represents [GMOA]/([GMOA]+[EtOA]).
